# Advances in the Detection and Identification of Bacterial Biofilms Through NIR Spectroscopy

**DOI:** 10.3390/foods14060913

**Published:** 2025-03-07

**Authors:** Cristina Allende-Prieto, Lucía Fernández, Pablo Rodríguez-Gonzálvez, Pilar García, Ana Rodríguez, Carmen Recondo, Beatriz Martínez

**Affiliations:** 1 Civil, Environmental and Geomatics Engineering Research Group (CEGE), Area of Cartographic, Geodesic and Photogrammetric Engineering, Department of Mining Exploitation and Prospecting, Polytechnic School of Mieres, University of Oviedo, 33003 Oviedo, Spain; 2 Dairy Products Institute of Asturias, C/Francisco Pintado Fe, 26, 33011 Oviedo, Spain; lucia.fernandez@ipla.csic.es (L.F.); anarguez@ipla.csic.es (A.R.); bmf1@ipla.csic.es (B.M.); 3 Department of Mining Technology, Topography and Structures, Universidad de León, Avda. Astorga s/n, 24401 Ponferrada, Spain; p.rodriguez@unileon.es; 4 DRACONES Research Group, Universidad de León, Avda. Astorga s/n, 24401 Ponferrada, Spain; 5 Remote Sensing Applications (RSApps) Research Group, Area of Cartographic, Geodesic and Photogrammetric Engineering, Department of Mining Exploitation and Prospecting, Polytechnic School of Mieres, University of Oviedo, 33003 Oviedo, Spain; mdrecondo@uniovi.es

**Keywords:** NIR, optical spectroscopy, bacterial biofilms, food safety

## Abstract

Bacterial biofilms play an important role in the pathogenesis of infectious diseases but are also very relevant in other fields such as the food industry. This fact has led to an increased focus on the early identification of these structures as prophylaxes to prevent biofilm-related contaminations or infections. One of the objectives of the present study was to assess the effectiveness of NIR (Near Infrared) spectroscopy in the detection and differentiation of biofilms from different bacterial species, namely *Staphylococcus epidermidis*, *Staphylococcus aureus*, *Enterococcus faecium*, *Salmonella Typhymurium, Escherichia coli*, *Listeria monocytogenes*, and *Lactiplantibacillus plantarum*. Additionally, we aimed to examine the capability of this technology to specifically identify *S. aureus* biofilms on glass surfaces commonly used as storage containers and processing equipment. We developed a detailed methodology for data acquisition and processing that takes into consideration the biochemical composition of these biofilms. To improve the quality of the spectral data, SNV (Standard Normal Variate) and Savitzky–Golay filters were applied, which correct systematic variations and eliminate random noise, followed by an exploratory analysis that revealed significant spectral differences in the NIR range. Then, we performed principal component analysis (PCA) to reduce data dimensionality and, subsequently, a Random Forest discriminant statistical analysis was used to classify biofilms accurately and reliably. The samples were organized into two groups, a control set and a test set, for the purpose of performing a comparative analysis. Model validation yielded an accuracy of 80.00% in the first analysis (detection and differentiation of biofilm) and 93.75% in the second (identification of biofilm on glass surfaces), thus demonstrating the efficacy of the proposed method. These results demonstrate that this technique is effective and reliable, indicating great potential for its application in the field of biofilm detection.

## 1. Introduction

The formation of bacterial biofilms poses substantial challenges in diverse environments, ranging from industrial systems to clinical settings and food contexts. Biofilms, structured bacterial communities enclosed in a self-produced polymeric matrix, exhibit heightened resistance to antibiotics, disinfectants, and environmental stressors. As a result, these structures have an impact on hygiene, product quality, and human health [1]. The economic repercussions of biofilms are significant, with an estimated annual cost exceeding $5 trillion USD across various industries, including food production, water security, and health [2].

The inherent threats associated with biofilm formation are particularly relevant when these communities are the reservoir of pathogenic bacteria. For instance, biofilm formation contributes to the colonization by and persistence of *Staphylococcus aureus* on diverse surfaces, including implant devices, human tissues and food-contact surfaces globally [3]. *Staphylococcus epidermidis*, an opportunistic bacterium, is also important in the human health context, being one of the most important microorganisms causing, among others, catheter-related infections due to its ability to adhere to biotic and abiotic surfaces [4]. *Enterococcus faecium* is often associated with multidrug-resistant nosocomial infections, being the main cause of infective endocarditis [5]. In terms of food safety, *Salmonella* species, *Escherichia coli* STEC, and *Listeria monocytogenes*, are amongst the most important foodborne pathogens recorded by the European Food Safety Authority (EFSA) as cause of important outbreaks in the EU. In contrast, biofilms formed by probiotic bacteria, such as *Lactiplantibacillus plantarum* are beneficial for prevention of some human diseases such as colonization with undesirable bacteria (i.e., urinary infections) [6].

In the food industry, the challenge of biofilm formation extends beyond product quality and safety, as biofilms are also considered responsible for damage to food processing equipment. Moreover, 80% of bacterial infections in the USA are believed to be specifically related to food-borne pathogens in biofilms [7]. This problem affects a wide range of food industries, including those involved in processing seafood, dairy, and meat, as well as those in the brewing sector. In this context, early detection is very important for the prevention of foodstuffs contamination and proper adherence to safety standards [8,9].

Various methods for detecting biofilms have been developed, encompassing different detection approaches to study bacterial biofilm formation and dynamics [10]. Among which ATP swabbing, contact plating and specific dyes for visual identification are the most commonly used in the food industry [11]. Moreover, surface sensors capable of detecting early-stage biofilms have been explored [12]. Additionally, innovative strategies to prevent biofilm formation are being explored to thwart bacterial attachment to surfaces [13].

In healthcare settings, where biofilm formation affects disinfection effectiveness and increases the risk of infections and antimicrobial resistance, continuous monitoring of critical surfaces is crucial. This facilitates the implementation of specific cleaning protocols, ultimately reducing the risk of healthcare-associated infections [14]. Disposable parts of some devices, such as ventilators and catheters, do not require after-use decontamination, but parts that can be in contact with a patient are required to be cleaned and disinfected with an approved product. Despite this, bacteria can remain within the device. Detection of biofilm presence and subsequent identification of specific bacteria becomes pivotal for the further optimization of antimicrobial strategies, especially given the importance of preventing antibiotic resistance spread in hospital environments [15].

Several spectroscopy techniques, including FTIR (Fourier Transform Infrared) spectroscopy and Raman spectroscopy, have proven to be effective for studying bacterial biofilms by providing detailed information on chemical profiles, matrix composition, and macrocomponents. These analyses are very useful for medical applications [16,17]. Also, near-infrared (NIR) spectroscopy has been previously studied for application in the food industry, indeed, it was particularly effective in real-time biofilm monitoring of beer canning lines, utilizing a microspectrometer paired with a halogen NIR light source [18]. The implementation of NIR stands out as an innovative approach compared to conventional infrared spectroscopy (FTIR) and Raman spectroscopy techniques. Unlike FTIR, NIR spectroscopy allows for deeper penetration into materials, providing more comprehensive information about the composition and structure of biofilms, which is crucial for analyzing biofilms on diverse food contact surfaces and within food matrices. Furthermore, unlike Raman spectroscopy, NIR spectroscopy is not limited by fluorescence, facilitating accurate detection in the complex biological samples often encountered in food products.

Our previous study on the use of NIR spectroscopy for biofilm detection [19] aimed to develop a rapid and efficient method for the early detection of biofilms formed by foodborne bacteria, including pathogens. Early detection is essential for implementing timely control measures and preventing the establishment of difficult-to-remove biofilms. This work builds upon our previous findings by optimizing the NIR technique and validating its performance with biofilms formed by various microorganisms on a range of relevant food contact materials. Thus, this study evaluates the effectiveness of NIR spectroscopy in detecting and differentiating biofilms formed by different bacterial species, relevant for the food industry and clinical settings, including *S. epidermidis*, *S. aureus*, *E. faecium*, *Salmonella* Typhymurium, *E. coli*, *L. monocytogenes*, and *L. plantarum*. To do that, we apply advanced spectral data analysis techniques, including Standard Normal Variate (SNV) and Savitzky–Golay (SG) filtering for preprocessing and Random Forest (RF) for classification. These methods offer advantages over traditional approaches like polynomial baseline correction and PLS regression, providing improved noise handling, nonlinearity management, and classification accuracy. Focusing on *S. aureus* biofilms, we further explored the effectiveness and versatility of NIR spectroscopy in precisely identifying biofilms formed by different strains on various surfaces, in order to test its application potential in different environments.

## 2. Materials and Methods

### 2.1. Sample Settings

The bacteria used in this study include *Salmonella enterica* subsp. *enterica* serotype *typhimurium* CECT 4594, *E. coli* CECT 434, *L. monocytogenes* CECT 935, *L. plantarum* 55-1 [20], *S. aureus* 15981 [21], *S. epidermidis* F12 [22] and *E. faecium* MMRA [23]. All strains were routinely grown in TSB (Tryptic Soy Broth, Scharlau, Barcelona, Spain) at 37 °C with shaking, except *L. plantarum* which was grown at 32 °C in MRS without shaking. Overnight cultures of these strains were diluted 1:100 in fresh TSB medium supplemented with 0.25% glucose (TSBg).

For the interspecies biofilm, 2 mL of each bacterial suspension were inoculated into 8-well polystyrene microtiter plates (Thermo Scientific, NUNC, Madrid, Spain). Seven wells were inoculated with each bacterium under study, while the eighth well was left as an uncontaminated control containing TSBg alone. This assay was performed in triplicate, analyzing a total of 24 samples (including bacterial and control samples). Four spectral signature measurements were recorded for each well, recording a total of 50 signatures per measurement, resulting in 96 measurements in total.

For *S. aureus* detection on glass, 2 mL of cell suspension were inoculated into each well of two-well µ-slides with a glass bottom (Ibidi GmbH, Martinsried, Germany). Four wells contained contaminated samples and two were inoculated with TSBg alone as a control. All samples were grown for 24 h at 37 °C allowing biofilm formation on the glass surface. After removing the planktonic phase, the attached cells were washed with 1 mL of PBS (138 mM NaCl, 30 mM KCl, 162 mM Na_2_HPO_4_, 30 mM KH_2_PO_4_, pH 7.4) prior to being scanned with the spectrophotometer.

After incubation and washing, six recordings were taken for each of them, resulting in a total of 36 sample measurements.

### 2.2. Spectral Data Acquisition

The acquisition of the spectral signatures of the different samples was carried out with an optical sensor, a LabSpec 4 Standard-Res, which is a portable analytical spectral device crafted by Analytical Spectral Devices (ASD, Boulder, CO, USA) [24]. It boasts a spectral resolution of 3 nm in the visible and near-infrared (VNIR) range, and 10 nm in the short-wave infrared (SWIR) range, enhancing its suitability for precise spectral analysis while offering mobility. Spectral information is acquired through contact measurements using a Contact Probe^®^ [25] with ATR cells, minimizing errors associated with stray light and enabling sample measurement through a transparent container. The ATR cells perform 50 reflections to capture the electromagnetic spectrum at various points within each sample. This yields a spectral signature, representing the sample’s spectral response for each wavelength in the analyzed region (350–2500 nm).

Prior to scanning each series of samples, we calibrated the device by using a white reference surface (Spectralon^®^) [26] a highly reflective material with a Lambertian reflectance profile. This reference surface serves as a baseline panel to ensure accurate spectral measurements.

### 2.3. Data Analysis

The spectral signatures obtained from the sampling process were subjected to SNV (Vector Normal Standardization) and Savitzky–Golay filters to improve data quality. The SNV filter eliminates variations in signal intensity due to factors such as light scattering, correcting systematic variations in spectral signatures. The Savitzky–Golay filter, a smoothing filter, removes random noise and unwanted fluctuations, resulting in better-defined peaks and valleys in the spectral signatures. Applying these filters enhances accuracy in biofilm detection and classification, improving data interpretation and facilitating the pattern identification.

NIR spectral data were then examined using principal component analysis (PCA), an unsupervised method commonly used in spectral signature analysis to enhance interpretation and enable the discovery of meaningful patterns [27]. The optimal number of principal components, those accumulating the highest variance, was determined and used in the Random Forest (RF) discriminant statistical analysis, a robust machine learning technique based on building multiple decision trees [28] suitable for this analysis due to its ability to handle large data sets, correlated predictor variables, and complex patterns, while avoiding overfitting and assessing variable importance. Specifically, we used a Random Forest model with 500 trees, applying the bootstrap method to train each tree with a randomly sampled subset of data and exploring the *mtry* parameter through cross-validation to optimize the number of features considered at each split and improve performance.

All the processing and analysis of spectral signatures was carried out using the statistical programming environment R (version 4.2.2) [29].

For model variation, samples were randomly divided into training and test set. Discriminant statistical models were established using the training set and validated with the test set, ensuring robust and applicable conclusions.

The results were evaluated using key statistical tools: the confusion matrix, the kappa coefficient, accuracy, precision, recall and F1-score. The confusion matrix visualizes the performance of a classification algorithm by comparing actual classifications with model predictions: true positives (TP), false positives (FP), true negatives (TN), and false negatives (FN). The kappa coefficient compares observed agreement with chance agreement [30], adjusting model accuracy by considering random matches.

Accuracy refers to the proportion of correct predictions among all cases. Precision measures the proportion of correct predictions against all positive predictions while recall assesses the proportion of correct positive predictions relative to all actual positive cases. The F1-score (1) harmonizes precision and recall into a single measure, calculated as the harmonic mean:

(1) $$F1\text{-}score=\frac{2\cdot \left(Precision\cdot Recall\right)}{\left(Precision+Recall\right)}$$

The choice of F1-score as a key metric is justified by its ability to assess classification performance in Vis-NIR spectroscopy [31,32].

These metrics provide valuable information about the quality and reliability of the statistical models applied in this study, thus allowing for a deeper and more accurate interpretation of the obtained results.

## 3. Results and Discussion

### 3.1. Biofilms Formed by Different Bacterial Strains

**Figure 1:**
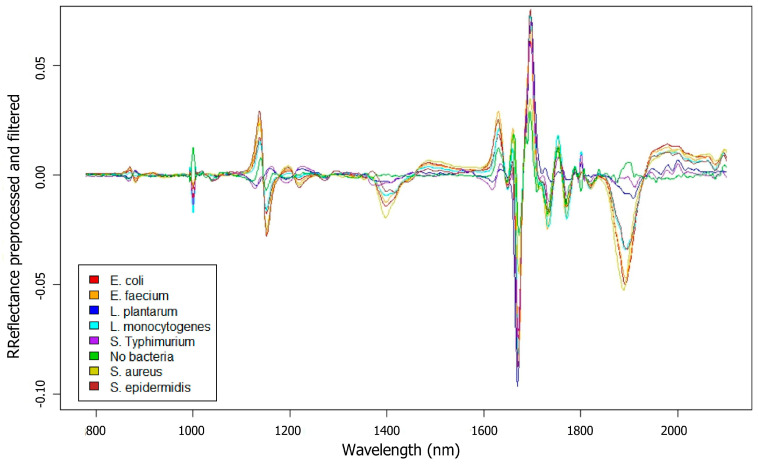
Spectral signatures obtained after NIR measurement of each bacterial biofilm. Bacterial species and control are indicated on the bottom left.

The spectral signatures of all analyzed samples, once averaged and filtered, were subjected to exploratory analysis. This analysis revealed that the most significant spectral differences between the samples lie within the NIR range, spanning from 780 to 2100 nm. The following figure (Figure 1) shows the spectral signatures averaged and preprocessed from the samples recorded for each biofilm type. Clear spectral differences suggest the potential of spectroscopy for differentiating between these bacterial species.

Near-infrared (NIR) spectroscopy allows differentiation of biofilms from the culture medium (TSBg) due to the interaction of NIR light with components of the extracellular matrix (polysaccharides, proteins, and nucleic acids), as well as the presence of water and glucose [19,33,34]. The higher reflectance values in biofilms, compared to the control (TSBg without bacteria), are attributed to the presence of these components, which affect the spectral properties.

The identification of biofilms formed by seven bacterial species adhered to a polystyrene surface was performed by measurement of 24 h-old biofilms. In this analysis, the samples were divided into two sets: a training set, comprising 60% of the samples for calibrating the statistical model to recognize patterns, and the remaining 40% forming a test set for model validation. Through the application of PCA, we managed to reduce the dimensionality of the data to 20 essential components, which were then used in the subsequent statistical modeling.

**Figure 2:**
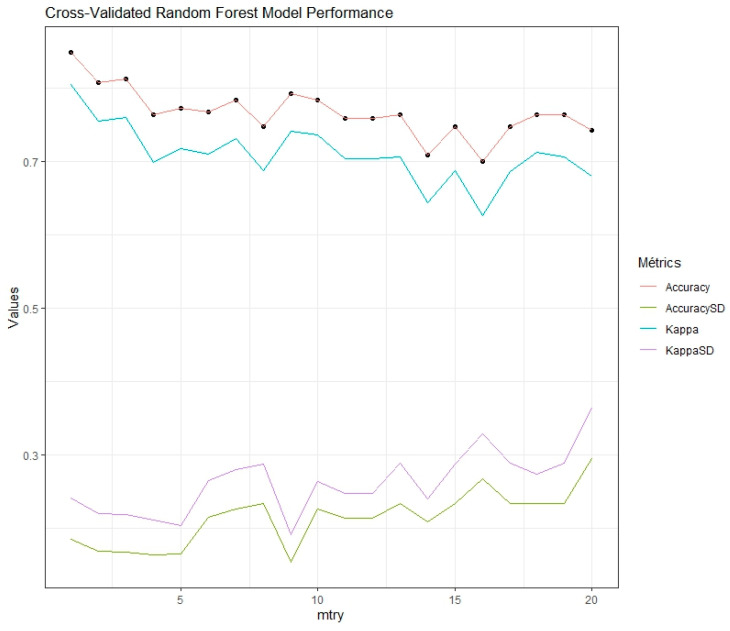
Random Forest performance: Influence of *mtry* on accuracy and stability.

We performed 10-fold cross-validation to robustly evaluate model performance. We explored mtry values ranging from 1 to 20 to optimize a Random Forest model trained with 500 trees. Figure 2 presents the resulting metrics (accuracy and Cohen’s Kappa), representing the average of the 10 cross-validation evaluations and their standard deviations to assess model stability. Even though mtry = 3 showed better average performance, we selected mtry = 9 because it displayed lower standard deviations, suggesting better generalization.

The validation results revealed a model accuracy of 80.00%, with a *p*-value < 0.05, indicating statistical significance. Furthermore, the kappa index reached a value of 0.7714, reflecting substantial agreement beyond chance in the classification performed by the model. The results obtained from the confusion matrix of the discriminant statistical analysis are shown in Table 1, in which columns represent the actual categories of the samples, that is, the confirmed classification of the bacteria, and the rows indicate the predictions made by the model based on NIR spectroscopy analysis.

**Figure 3:**
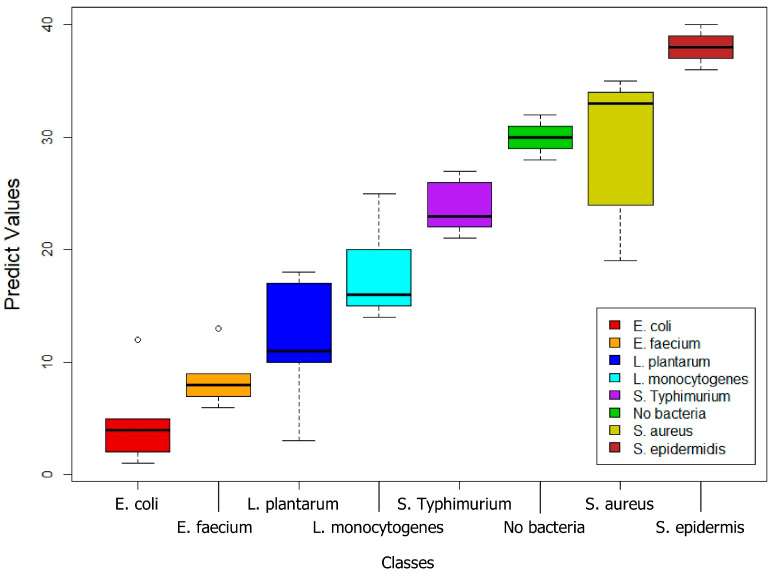
Distribution of the bacterial samples.

In Figure 3, the boxplot provides a visual summary of the data distribution. The central boxes depict the interquartile range, stretching from the first quartile (Q1) to the third quartile (Q3), with a line indicating the median (Q2). The whiskers extend to the maximum and minimum values within a calculated range, typically 1.5 times the interquartile range from the quartiles, and points outside these whiskers are considered outliers, highlighting exceptional variations. On the *X*-axis, the different bacterial species are categorized, the *Y*-axis displays the model prediction values assigned by the RF model to each sample within the predicted bacterial class. This visualization provides insights into the centrality, dispersion, and potential outliers within the data distribution for each bacterial class, revealing variations across species and indicating differences in their measurable characteristics.

**Figure 4:**
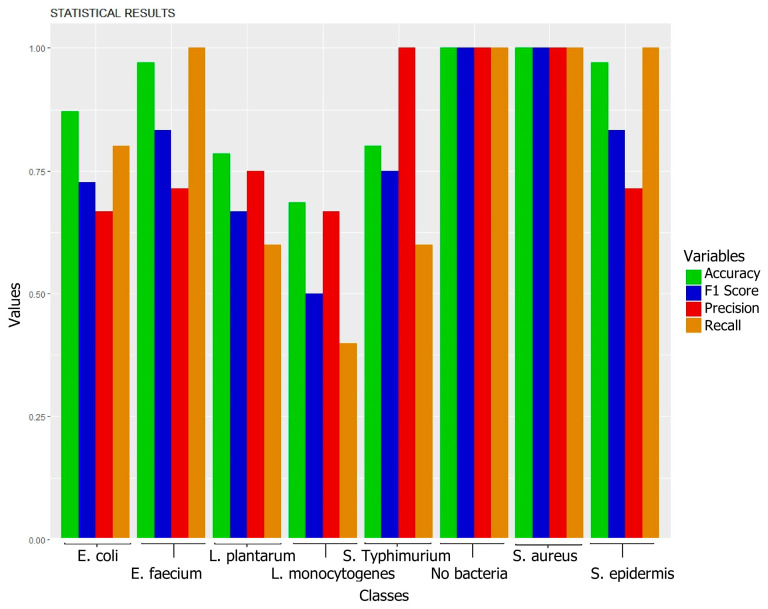
Performance metrics of the Random Forest model.

Figure 4 displays the results of the discriminative statistical analysis using the Random Forest algorithm. The data depicted in the bar chart reflect the key performance metrics of the model: accuracy, precision, recall, and F1-score. Each bar in the chart represents one of these metrics, allowing for an immediate visual comparison among them and facilitating the interpretation of the model’s overall effectiveness. The height of the bars reflects the percentage value of each metric, where the value of 1 represents perfect performance for the corresponding metric.

The evaluated statistical model exhibits variability in its capacity to classify different types of bacteria. For *E. coli*, despite a good overall performance (80% recall, Table 1), precision was 66.7% (Figure 3) due to the misclassification of *L. plantarum* (40%) and *L. monocytogenes* (20%) as *E. coli* (Table 1). This confusion could be attributed to the presence of shared components in multi-species biofilms, a common challenge in real-world environments such as those described in the introduction for the food industry, where the coexistence of different species is frequent. For *L. plantarum*, performance was moderate (60% recall, 75% precision, 78.6% accuracy; Table 1, Figure 3), with 20% of *E. coli* samples misclassified as *L. plantarum* (Table 1), resulting in an F1-score of 0.67 (Figure 3). The prediction distribution for *L. plantarum* is shown in Figure 2. As with *E. coli*, the confusion could be due to shared components, highlighting the need for future research to improve the method’s specificity, crucial for food quality control applications where differentiating between these microorganisms is essential. For *E. faecium*, perfect recall (100%, Table 1) and high accuracy (97.1%, Figure 3) were achieved. However, the model misclassified 40% of *L. monocytogenes* samples as *E. faecium* (Table 1), reducing precision to 71.4% (Figure 3). The prediction distribution is shown in Figure 2. Despite this limitation, the F1-score remained high (0.83, Figure 3). Considering the importance of *E. faecium* in multidrug-resistant nosocomial infections, based on these results, NIR spectroscopy presents itself as a promising tool for its rapid identification, although optimization of differentiation with other species, especially *L. monocytogenes*, is required. For *L. monocytogenes*, performance was the lowest (40% recall, Table 1), with frequent confusion with *E. faecium* (40%) and *S. typhimurium* (20%) (Table 1), resulting in 66.7% precision and a low F1-score (0.50, Figure 4). The 68.6% accuracy (Figure 4) and the prediction distribution (Figure 3) highlight the need to improve the model’s ability to identify this pathogen. Given its relevance in foodborne outbreaks, optimizing the model for *L. monocytogenes* is crucial to ensure food safety. For *S. typhimurium*, 60% recall (Table 1) and 100% precision (Figure 4) were observed, despite 20% of *S. epidermidis* samples being misclassified as *S. typhimurium* (Table 1). This resulted in an F1-score of 0.75 and 80% accuracy (Figure 4). The prediction distribution is shown in Figure 3. Like *L. monocytogenes*, *S. typhimurium* is a relevant foodborne pathogen, so improving its detection through NIR spectroscopy could have a significant impact on outbreak prevention. For *S. aureus* and *S. epidermidis*, the model perfectly identified *S. aureus* samples (100% across all metrics; Table 1, Figure 4). This result is particularly relevant given the importance of *S. aureus* in the contamination of various surfaces, including medical devices, human tissues, and food contact surfaces, as highlighted in the introduction. *S. epidermidis* showed good performance with 100% recall (Table 1), but the misclassification of 20% of *S. typhimurium* samples as *S. epidermidis* reduced precision to 71.4% (Figure 4). The F1-score was 0.83 and accuracy was 97.1% (Figure 4). The prediction distribution is shown in Figure 3. The high sensitivity for *S. epidermidis* is promising, considering its implication in catheter-related infections.

### 3.2. Biofilms Formed by S. aureus on Glass

**Figure 5:**
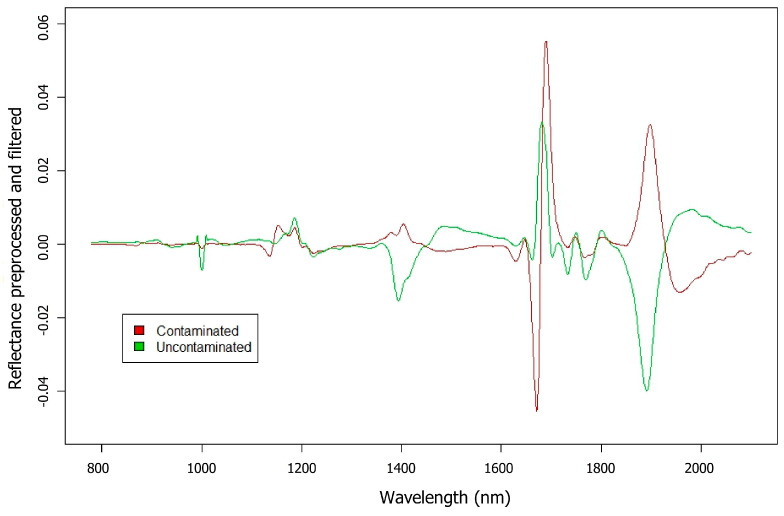
Average spectral signatures of contaminated and uncontaminated samples.

In order to assess NIR spectroscopy for the identification of *S. aureus* adhered to glass surfaces, 24 h-old biofilms formed by *S. aureus* 15981 were obtained and quantified. Also, an uncontaminated glass plate was used for control proposes. In this analysis, both contaminated and uncontaminated samples were placed in glass wells. Figure 5 displays the averaged spectral signatures of the samples with reflectance values resulting from preprocessing as a preliminary exploratory analysis. The results revealed significant differences across various wavelengths between contaminated and uncontaminated samples. These differences indicate unique features in the spectral signatures that are crucial for their classification.

**Figure 6:**
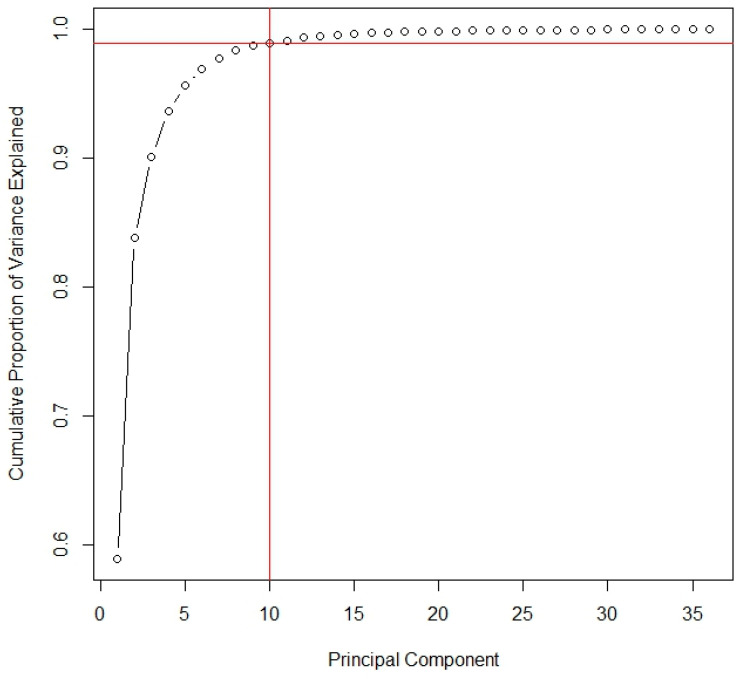
Principal component analysis: cumulative variance explained.

For RF analysis, all samples were also divided into two sets, training and test, following a 60–40% ratio, respectively. The PCA technique allowed for the reduction of variables to a total of 10 main components. Figure 6 shows the results of the principal component analysis. This figure displays the cumulative proportion of variance explained by the principal components. The red lines highlight the selected number of components (10), which explain 98.95% of the total variance. This selection, based on the elbow point in the scree plot, captures nearly all of the variance while minimizing the number of components.

Once the cross-validation analysis was performed to determine the optimal value of the mtry parameter, the results showed perfect stability starting from *mtry* = 3, achieving an accuracy of 0.96, with a standard deviation of accuracy of 0.0894, a Kappa of 0.9091, and a standard deviation of Kappa of 0.2033. Therefore, it was determined that *mtry* = 3 was the optimal value to configure the Random Forest model. Once the model was defined using the training set, it was validated with the test set. Results showed an accuracy of 95.45%, a precision of 83.33%, a recall of 100%, and an F1-score of 90.91%, along with a kappa index of 0.8621. These outcomes do not only reflect the model’s high capability to correctly classify samples, but also highlight its efficiency in identifying positive cases, as indicated by the perfect recall. Precision, although slightly lower than recall, remains robust and suggests that the proportion of true positives over all instances classified as positive is high. The balance between precision and recall is captured in the F1-score, which harmonizes both metrics and provides an integrated measure of the model’s accuracy. The kappa index, measuring agreement beyond chance, corroborates the model’s soundness. The statistical significance of the results, indicated by a *p*-value < 0.05, suggests a real classification ability of the model, reinforcing the reliability and reproducibility of the model under different experimental conditions. The results obtained from the confusion matrix of the discriminant statistical analysis are shown in Table 2, where the columns represent the actual classification of the bacteria and the rows, the model’s predictions:

This study demonstrates the potential of NIR spectroscopy for detecting *S. aureus* biofilms on glass, a critical issue for medical devices and food contact surfaces.

## 4. Conclusions

NIR spectroscopy, combined with multivariate classification, has proven to be promising in the detection and differentiation of bacterial biofilms. The decision to apply Near-Infrared (NIR) spectrometry techniques as the main focus of our research is based on the search for a highly efficient and precise option for the early detection of biofilms. A key innovation in this study is the integration of preprocessing techniques like SNV and Savitzky–Golay filters with advanced statistical methods such as Random Forest, allowing for the effective handling of large and complex datasets. This approach not only enhances detection accuracy but also represents an advancement compared to more traditional methods.

The analyses have shown that the technique is effective and reliable, indicating great potential for its application in the field of biofilm detection formed on different materials. From the evaluated statistical models, some variability is observed in their capacity to classify different types of bacteria, with generally good results for all bacteria except for *L. monocytogenes*, which shows lower performance, particularly in terms of sensitivity, suggesting that the model could be refined for more effective detection of these bacteria.

On the other hand, the precise identification of *S. aureus* is of great importance given its relevance both in the food industry and in the clinical environment. Our findings demonstrate excellent classification results for this pathogen, highlighting the practical applications of this technique in preventing biofilm-related infections and contamination of surfaces. The ability to effectively detect and classify this pathogen underscores the practical value of the NIR technique, which could have significant implications in the prevention and control of infections related to biofilms.

NIR spectroscopy is a fast and non-destructive technique, meaning that samples can be analyzed without alteration, maintaining their integrity for future use or continuous analysis. Moreover, NIR is less affected by the presence of water, which is advantageous when studying biological systems or processes where water is a predominant component. This contrasts favorably with other methods like FTIR or Raman spectroscopy, which are more sensitive to water interference. Sample preparation is simpler, as it does not require controlled environmental conditions or special treatments. This technique also allows for real-time analysis, facilitating the monitoring and control of industrial processes instantly. Lastly, from an operational cost perspective, NIR equipment tends to be less expensive to maintain and operate compared to Raman or FTIR equipment, representing an additional economic advantage.

While these advantages make NIR spectroscopy a promising tool for biofilm detection, it is important to recognize that applying the method under more diverse conditions requires more precise calibration. Our focus on a specific group of bacteria and controlled laboratory conditions allowed us to demonstrate the feasibility of the technique. To address this limitation and broaden the applicability to larger-scale studies, future research will focus on combining NIR spectroscopy with imaging techniques. This combined approach will not only allow for the analysis of larger and more representative surface areas but also enable us to evaluate the efficacy of this technique for detecting biofilm formation on various food-industry relevant materials, such as stainless steel, polyethylene, etc. To further broaden the scope of this work, future studies will also address calibration and application under a broader range of conditions and replicate the analyses on a wider variety of surfaces. This will reinforce the reliability of the results obtained and expand the applicability of the method to different contexts and materials. Adaptability and cross-validation in various conditions and surfaces will establish NIR spectroscopy as a standard tool in biofilm detection, paving the way for its integration into routine monitoring protocols and early warning systems.

## Figures and Tables

**Table 1:** Confusion matrix resulting from the analysis of biofilms formed by different bacteria.

	*E. coli*	*E. faecium*	*L. plantarum*	*L. monocytogenes*	*S. typhimurium*	Control	*S. aureus*	*S. epidermidis*
*E. coli*	80.00%	0.00%	40.00%	20.00%	0.00%	0.00%	0.00%	0.00%
*E. faecium*	0.00%	100.00%	0.00%	40.00%	0.00%	0.00%	0.00%	0.00%
*L. plantarum*	20.00%	0.00%	60.00%	0.00%	0.00%	0.00%	0.00%	0.00%
*L. monocytogenes*	0.00%	0.00%	0.00%	40.00%	20.00%	0.00%	0.00%	0.00%
*S. typhimurium*	0.00%	0.00%	0.00%	0.00%	60.00%	0.00%	0.00%	0.00%
Control	0.00%	0.00%	0.00%	0.00%	0.00%	100.00%	0.00%	0.00%
*S. aureus*	0.00%	0.00%	0.00%	0.00%	0.00%	0.00%	100.00%	0.00%
*S. epidermidis*	0.00%	0.00%	0.00%	0.00%	20.00%	0.00%	0.00%	100.00%

**Table 2:** Confusion matrix resulting from the analysis of biofilms obtained from contaminated and uncontaminated samples.

	*Contaminated*	*Uncontaminated*
*Contaminated*	100.00%	9.09%
*Uncontaminated*	0.00%	90.91%

## Data Availability

The original contributions presented in the study are included in the article, further inquiries can be directed to the corresponding author.
